# Epidemiological characteristics and trends of a Nationwide measles outbreak in Mongolia, 2015–2016

**DOI:** 10.1186/s12889-019-6511-0

**Published:** 2019-02-15

**Authors:** Oyunchimeg Orsoo, Yu Mon Saw, Enkhbold Sereenen, Buyanjargal Yadamsuren, Ariunsanaa Byambaa, Tetsuyoshi Kariya, Eiko Yamamoto, Nobuyuki Hamajima

**Affiliations:** 1 Department of Healthcare Administration, Nagoya University Graduate School of Medicine, 65 Tsurumai-cho, Showa-ku, Nagoya 466-8550 Japan; 2 Department of Medical Service, Ministry of Health, Ulaanbaatar, Mongolia; 3 Nagoya University Asian Satellite Campuses Institute, Nagoya, Japan; 4 Department of Public Administration and Management, Ministry of Health, Ulaanbaatar, Mongolia; 5 Department of Microbiology and Immunology, School of Pharmacy and Bio-Medicine, Mongolian National University of Medical Sciences, Ulaanbaatar, Mongolia

**Keywords:** Measles, Outbreak, Reported cases, Elimination, Vaccination, Mongolia

## Abstract

**Background:**

Mongolia was one of the four countries that received a measles-elimination certificate from the World Health Organization Regional Office for the Western Pacific in 2014. Following the outbreaks in many countries including China, a large measles outbreak occurred in Mongolia in 2015. This study reports 2015–2016 measles outbreak incidence, mortality, and complications, according to time, geographical distribution, and host characteristics.

**Methods:**

The epidemiological characteristics and trends of measles outbreak were analyzed using the Mongolian national surveillance data reported to the Center for Health Development, Ministry of Health, from January 2015 to December 2016.

**Results:**

In total, 23,464 cases of measles including eight deaths were reported in 2015, and 30,273 cases of measles including 132 deaths were reported in 2016, which peaked in June 2015 and March 2016, respectively. Majority of the cases were reported from Ulaanbaatar (35,397, 65.9%). The highest attack rates were 241 per 10,000 population in Darkhan-Uul aimag, and 263 per 10,000 population in Ulaanbaatar. Measles-related death, nosocomial infection, and complications were most frequent among children aged < 1 year.

**Conclusions:**

Following no reports of measles since 2011, a large nationwide outbreak occurred in Mongolia, despite the high vaccination coverage in the past. The highest incidence rate was reported in Ulaanbaatar city, and Umnugovi aimag in 2015 and Darkhan-Uul aimag in 2016. The most affected age group were aged < 1 year and those aged 15–24 years. Mortality cases were prominent among children aged < 1 year who were not eligible for vaccination. A systematic vaccination strategy is required to prevent another measles outbreak.

**Electronic supplementary material:**

The online version of this article (10.1186/s12889-019-6511-0) contains supplementary material, which is available to authorized users.

## Background

Measles is one of the leading causes of death among young children in developing countries despite the availability of a safe and effective vaccines [1]. It also causes severe complications, particularly among children, and includes pneumonia, encephalitis, keratitis, and otitis media [2, 3]. Immunity against measles is acquired by infection and usually lasts throughout life [4]. The World Health Organization (WHO) recommended a two-dose vaccination policy, with the first dose administered during the first year of life [5] and the coverage to be maintained at a level of at least 90–95% to interrupt the disease transmission [6].

In many countries, measles vaccine is included in the country’s immunization program and is freely available to all. Measles is considered as potentially eliminable disease because the reservoir is exclusively human, and sensitive and specific diagnostic tests, as well as safe effective vaccines, are available [7]. Globally, the annual incidence of measles decreased by 75.0% from 146 to 36 cases per million population during 2000–2015, and the annual estimated measles deaths decreased by 79.0% from 651,600 to 134,200 [8]. Recent outbreaks have been reported in North America [9, 10], South America [11], Europe [12, 13], Africa [14], and Asia [15–17].

In Mongolia, the first measles case was reported in 1957. During 1960–1972, the incidence of measles was 125 per 10,000 population. Measles accounted for 33.0% of all reported communicable diseases, and 32% of deaths were caused by infectious diseases. Mongolia introduced the Expanded Program on Immunization (EPI) in 1962 [18], but measles was only included in the EPI of children aged between eight and 11 months in 1973 with a one-dose measles containing vaccine (MCV). The two-dose vaccine (MCV2) was introduced [19] to children aged between 12 and 23 months, which was replaced later in 2009 with a measles–mumps–rubella vaccine (MMR). In September 2009, MMR was introduced and immunization schedule has switched to first dose MMR at nine months and second dose MMR at two years. Since 1994, the periodic measles supplemental immunization activities were carried out across the country, including each birth cohort born after 1985.

Mongolia maintained high measles vaccination coverage at > 95% since 2005. After introducing MCV2, measles cases gradually decreased during 1990–2002. However, three measles outbreaks were reported in 1992, 1995 and 2000–2002 [20]. After 2011, no measles case reported in Mongolia. In 2014, the WHO Regional Office for the Western Pacific (WPRO) certified measles elimination in Mongolia [21].

In March 2015, one year after the declaration of measles elimination by the WPRO, the first measles case was reported in Ulaanbaatar city. This case was overshadowed by a large outbreak that continued for approximately two years, and involving 53,737 cases and 140 deaths. This study aimed to describe the details of measles outbreak in Mongolia during 2015–2016. Although the government reports on this outbreak were available in Mongolian language and partly in English, this article provides findings from more detailed analysis of the national surveillance database, with the permission from the Ministry of Health, Mongolia.

## Methods

### Data sources

**Fig. 1:**
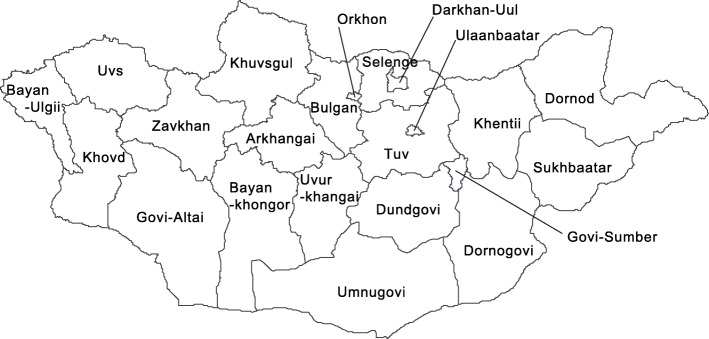
Map of Mongolia. Mongolia consists of 21 aimags and Ulaanbaatar city

According to the National Statistical Office of Mongolia, the estimated population of Mongolia was 3.1 million people in 2016. Majority (68.0%) of the population lived in cities, and the remainder resided in the rural areas in 2015 [22]. The administrative units of Mongolia are Ulaanbaatar city and 21 provinces known as aimags (Fig. 1). Data on measles cases from January 2015 to December 2016 were obtained from the database of the Center for Health Development (CHD) of Mongolia.

The database is based on the primary data collected by the National Center for Communicable Diseases from medical facilities as a part of the routine national monitoring system. Data comprised the demographic characteristics (age, sex, educational level, employment status, and patients’ address), and clinical information (complications, hospitalization, relation to nosocomial infection, admission date, date of onset of rashes, date of confirmation of diagnosis, date that the disease was reported, and vaccination status). The measles cases from March 2015, when the first measles case was reported, to December 2016 were included in this study. The population data were obtained from the CHD (Additional file 1).

### Case definition

The Mongolian National Surveillance System applies the WHO-recommended case definition. All measles cases in this study were clinically and/or laboratory-confirmed cases. A clinically confirmed case is defined as “any person in whom a clinician suspects measles infection, or any person with fever and maculopapular rash (i.e. non-vesicular) and cough, coryza (i.e. runny nose) or conjunctivitis (i.e. red eyes)” [23]. A laboratory confirmed case is defined as anyone who was positive for measles-specific IgM antibodies or measles RNA according to the reverse transcription polymerase chain reaction (RT-PCR). The vaccination status was generally reported by medical doctors who referred to the vaccination booklet for patients aged < 15 years and verbally asked about their vaccination status for patients aged ≥15 years. Persons with one or two doses vaccination history were defined as “vaccinated”, those with zero dose and unknown vaccination status were considered as “not vaccinated”.

The information of measles complications classified according to the International Classification of Diseases 10th revision was recorded at the time of diagnosis and death if the person died (Table 1). The characteristics of measles-related death (*n* = 140) were evaluated. To compare the death and survival cases, 120 out of 140 death cases were included in analysis due to missing data and difficultly in identification of death case among 53,737 cases.

**Table 1:** Complications of measles

Category	ICD-10 code
Measles complicated by encephalitis	B05.0
Measles complicated by meningitis	B05.1
Measles complicated by pneumonia	B05.2
Measles complicated by otitis media	B05.3
Measles with intestinal complications	B05.4
Measles with other complications	B05.8
Measles without complication	B05.9

### Statistical analysis

The analyses were performed using the Statistical Package for the Social Sciences (SPSS) version 24.0 (IBM SPSS Inc.). The maps and graphs were created using Adobe Illustrator CC and Microsoft Excel 2013, respectively. Mann-Whitney U test was applied to compare differences between two groups. A logistic regression model was performed to estimate the odds ratios (OR) and 95% confidence interval (CI). Differences were considered to be statistically significant if the *P-* value was < 0.05.

## Results

**Fig. 2:**
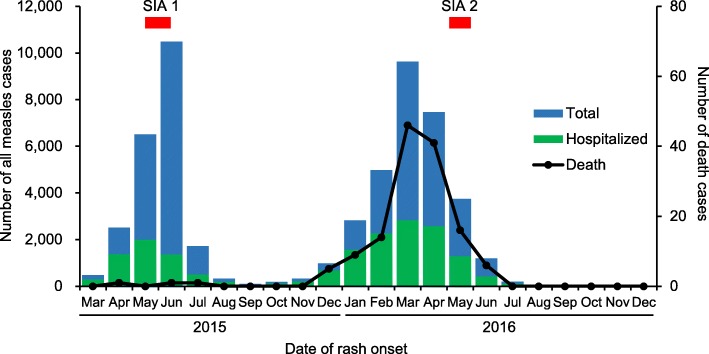
The incidence of measles in Mongolia, from March 2015 to December 2016. The incidence of all reported measles cases in the crude number is shown according to the date of rash onset, from March 2015 to December 2016. The number of death cases is shown on the right axis according to the date of death. SIA: Supplementary immunization activity

**Fig. 3:**
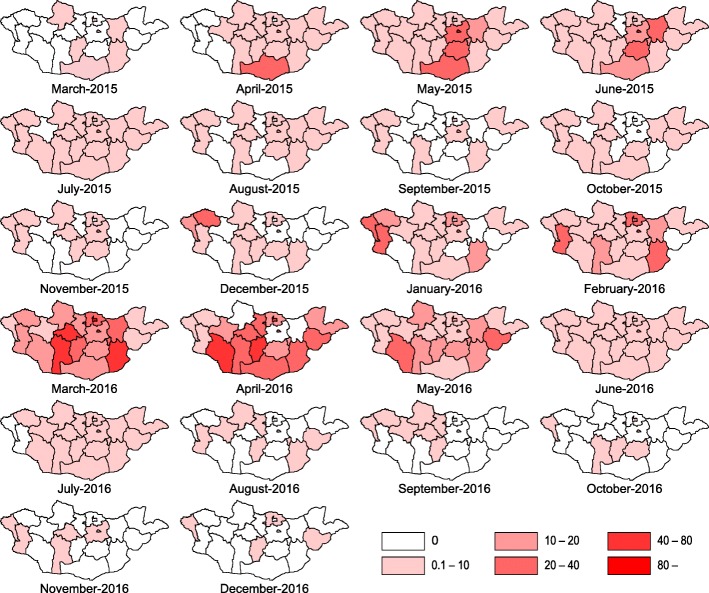
Geographical distribution of measles in Mongolia, from March 2015 to December 2016, The attack rate per 10,000 population in each aimag is categorized into 0, 0.1–10, 10–20, 20–40, 40–80, > 80, from March 2015 to December 2016

**Fig. 4:**
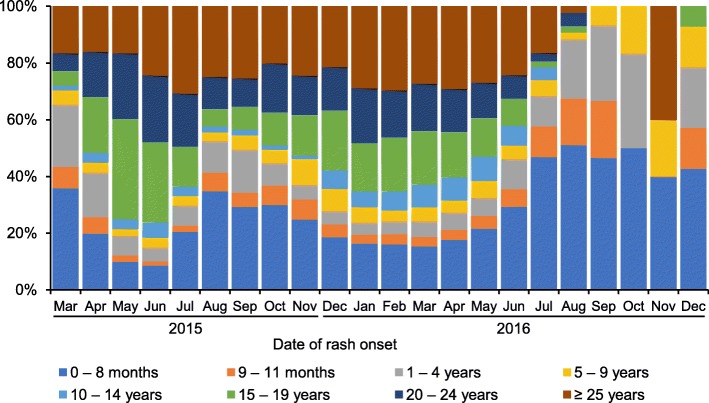
Age distribution of measles in Mongolia, from March 2015 to December 2016

**Fig. 5:**
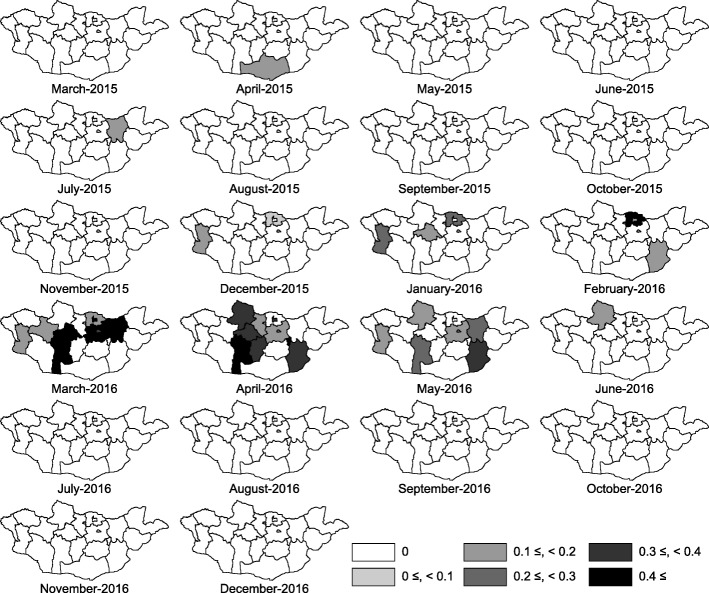
Geographical distribution of death cases due to measles in Mongolia, from March 2015 to December 2016. The mortality rate per 10,000 population in each aimag is shown, from March 2015 to December 2016

The first measles case was reported in March 18, 2015 in Ulaanbaatar city, where a population of 1.3 million (45.6% of the national population) reside. The first peak in the outbreak occurred in June 2015 and the second peak in March 2016. The outbreak declined in July 2016, although sporadic cases occurred in Ulaanbaatar city and several aimags thereafter. The number of confirmed measles cases was 53,737 from March 2015 to December 2016 (23,464 in 2015, and 30,273 in 2016). Clinically- laboratory-confirmed measles cases accounted for 95.6 and 4.4%, respectively. The attack rate was 180 per 10,000 population, and 33.0% of all patients with measles were hospitalized (Table 2, Figs. 2 and 3). The most affected age group were children aged < 1 year (18.9%), 15–19 years (18.9%), and 20–24 years (18.2%) [Fig. 4]. The median age (interquartile range, IQR) of all patients with measles was 19.0 (4.6–25.0) years throughout this outbreak, and the median (IQR) age in 2016 (18.5 [2.5–25.8]) became significantly lower than that in 2015 (19.2 [8.2–23.8])) (*P* < 0.001). The proportion of cases in children aged < 1 year increased in 2016. A total of 140 deaths cases were reported (eight in 2015 and 132 in 2016), and the death rate was 0.47 per 10,000 population. Majority of the reported cases occurred among children aged < 1 year. Death rate in children aged < 1 year was as high as 15.7 per 10,000 population of their corresponding age group. The number of hospitalized cases, death cases, and nosocomial infection in children aged < 1 year was significantly higher than all the other age groups (*P* < 0.001) [Tables 2 and 5, Fig. 5]. Only 6.5% of all measles cases had vaccination history of one or two doses. The majority had no history of vaccination.

**Table 2:** Demographic characteristics of measles outbreak in Mongolia, March 2015–December 2016 (*N* = 53,737)

	Total	Attack rate ^a^	Hospitalized	Death	Death rate ^a^	Vaccinated ^c^	Nosocomial infection
	*N* (%)		*n* (%) ^c^	*n* (%)		*n* (%) ^d^	*n* (%) ^d^
Gender							
Male	26,853 (50.0)	182	8843 (32.9)	80 (57.1)	0.54	1773 (6.6)	572 (2.1)
Female	26,884 (50.0)	177	8893 (33.1)	60 (42.9)	0.40	1699 (6.3)	530 (2.0)
Age							
0–8 months	8384 (15.6)	1278 ^b^	4881 (58.2) ^e^	105 (75.0) ^f^	15.7 ^b^	0 (0)	625 (7.5) ^g^
9–11 months	1769 (3.3)		966 (54.6) ^e^	20 (14.3) ^f^		313 (17.7)	151 (8.5) ^g^
1–4 years	3440 (6.4)	113	1385 (40.3)	9 (6.4)	0.29	897 (26.1)	110 (3.2)
5–9 years	2265 (4.2)	76	665 (29.4)	0 (0.0)	0	489 (21.6)	11 (0.49)
10–14 years	3323 (6.2)	153	531 (16.0)	0 (0.0)	0	546 (16.4)	7 (0.21)
15–19 years	11,383 (21.2)	482	2402 (21.1)	1 (0.7)	0.042	583 (5.1)	28 (0.25)
20–24 years	9763 (18.2)	379	2374 (24.3)	0 (0.0)	0	284 (2.9)	65 (0.67)
≥ 25 years	13,410 (25.0)	84	4532 (33.8)	5 (3.6)	0.031	360 (2.7)	105 (0.78)
All age	53,737 (100)	180	17,736 (33.0)	140 (100)	0.47	3472 (6.5)	1102 (2.1)

^a^Per 10,000 population of corresponding gender or age group as of 31st December, 2015

^b^Rate in under-one children

^c^Vaccination of one or two doses

^d^Percentage to total number of corresponding gender or age group

^e, f, g^The number of hospitalized cases, death cases, and nosocomial infection in under-one age were significantly higher than all the other age groups (*P* < 0.001). OR and 95% CI were 3.6 (3.5–3.8), 28.8 (17.0–48.8) and 10.8 (9.5–12.4), respectively

The measles cases were reported in all aimags and capital city of Ulaanbaatar. Ulaanbaatar city had the highest number of cases, and Ulaanbaatar and Darkhan-Uul had the highest attack rate throughout this outbreak in 2015–2016. However, the measles-related death rate in Ulaanbaatar was not as high as compared to its high attack rate (Table 3). Umnugovi, Dundgovi, Tuv and Khentii had a high attack rate in first peak of 2015. In the beginning of the second peak, the western (Bayan-Ulgii, Khovd and Uvs) and northern regions (Selenge) appeared to have a high attack rate, and measles spread to all over Mongolia (Fig. 3).

**Table 3:** Geographical characteristics of measles outbreak in Mongolia, March 2015–December 2016 (*N* = 53,737)

Residence	Total cases	Attack rate ^a^	Death cases	Death rate ^a^
Ulaanbaatar	35,397	263	54	0.40
Arkhangai	1087	119	10	1.1
Baynkhongor	1058	127	10	1.2
Bayn-Ulgii	669	68	0	0.0
Bulgan	273	46	1	0.17
Darkhan-Uul	2357	241	8	0.82
Dornod	433	57	0	0.0
Dornogovi	1176	186	5	0.79
Dundgovi	473	107	0	0.0
Govi-Altai	673	120	0	0.0
Govi-Sumber	184	113	1	0.61
Khentii	1004	139	8	1.1
Khovd	878	106	5	0.60
Khuvsgul	1025	80	11	0.86
Orkhon	1029	104	4	0.41
Selenge	1289	123	11	1.1
Sukhbaatar	526	90	0	0.0
Tuv	865	96	6	0.67
Umnugovi	808	132	1	0.16
Uvs	689	86	0	0.0
Uvurkhangai	1608	144	4	0.36
Zavkhan	236	34	1	0.14
Total	53,737	180	140	0.47

^a^Per 10,000 population in each Aimag as of 31st December, 2015

A total of 1009 cases had complications and 52,728 cases had no complications (Table 4). Complications were most common among children aged one year. Aside from the “other complications,” pneumonia was the most common complication of measles (*n* = 334, 33.1%). Pneumonia complication in children aged < 1 year was 52.4% (*n* = 175) in all age groups with pneumonia complication (*n* = 334), and 48.2% (*n* = 175) of all complications in children aged < 1 year was pneumonia while 24.6% (*n* = 159) was so in other age groups.

**Table 4:** Complications of reported measles cases according to age, March 2015–December 2016 (*N* = 53,737)

	Encephalitis	Meningitis	Pneumonia	Otis media	Intestinal complications	Other complications	Without complications	Total
	*n* (%)	*n* (%)	*n* (%)	*n* (%)	*n* (%)	*n* (%)	*n* (%)	*N*
Gender								
Male	15 (42.0)	1 (50.0)	171 (51.0)	1 (25.0)	19 (56.0)	271 (45.0)	26,375 (50.0)	26,853
Female	21 (58.0)	1 (50.0)	163 (49.0)	3 (75.0)	15 (44.0)	328 (55.0)	26,353 (50.0)	26,884
Age								
0–8 months	7 (19.4)	0 (0)	153 (45.8)	2 (50.0)	5 (14.7)	147 (24.5)	8070 (15.3)	8384
9–11 months	2 (5.6)	0 (0)	22 (6.6)	0 (0)	1 (2.9)	24 (4.0)	1720 (3.3)	1769
1–4 years	1 (2.8)	0 (0)	19 (5.7)	0 (0)	3 (8.8)	33 (5.5)	3384 (6.4)	3440
5–9 years	0 (0)	0 (0)	11 (3.3)	0 (0)	1 (2.9)	26 (4.3)	2227 (4.2)	2265
10–14 years	3 (8.3)	0 (0)	11 (3.3)	0 (0)	0 (0)	26 (4.3)	3283 (6.2)	3323
15–19 years	5 (13.9)	0 (0)	16 (4.8)	0 (0)	2 (5.9)	42 (7.0)	11,318 (21.5)	11,383
20–24 years	2 (5.6)	0 (0)	16 (4.8)	1 (25.0)	4 (11.8)	71 (11.9)	9669 (18.3)	9763
≥ 25 years	16 (44.4)	2 (100)	86 (25.7)	1 (25.0)	18 (52.9)	230 (38.4)	13,057 (24.8)	13,410
All age	36 (100)	2 (100)	334 (100)	4 (100)	34 (100)	599 (100)	52,728 (100)	53,737

The risk factors for death included aged < 1 year, residence outside Ulaanbaatar city, complications at the time of incidence reports were made, and nosocomial infection (Table 5).

**Table 5:** Characteristics of measles-related death cases and risk factors for death in Mongolia, March 2015–December 2016 (*N* = 53,737)

	Death	Survival	Unadjusted	Adjusted ^e^
	n or Median (IQR)	n or Median (IQR)	OR (95% CI)	OR (95% CI)
Total	120	53,617		
Gender				
Male	70	26,783	1.4 (0.98–2.0)	
Female	50	26,834	Ref	
Age (years)	0.56 (0.39–0.75)	19.0 (4.8–25.0) ^d^		
< 1	104	9872	28.8 (17.0–48.8)***	25.0 (14.7–42.6)***
≥ 1	16	43,745	Ref	Ref
Residence				
Ulaanbaatar	43	35,354	0.29 (0.20–0.42)***	0.34 (0.23–0.51)***
Other Aimags	77	18,263	Ref	Ref
Complication ^a^				
Complications ^b^	13	996	6.4 (3.6–11.5)***	2.2 (1.2–4.0)**
No complications ^c^	107	52,621	Ref	Ref
Route of transmission				
Nosocomial infection	11	1091	4.9 (2.6–9.1)***	2.1 (1.1–3.9)**
Others	109	52,526	Ref	Ref

** *P* < 0.01, *** *P* < 0.001; IQR, interquartile range; OR, odds ratio; CI, confidence interval

^a^Complications at the time of incident report

^b^B05.0, B05.1, B05.2, B05.3, B05.4, B05.8

^c^B05.9

^d^*P* < 0.001 (Mann-Whitney U test)

^e^Adjusted by age, region, complication, and route of transmission

**Fig. 6:**
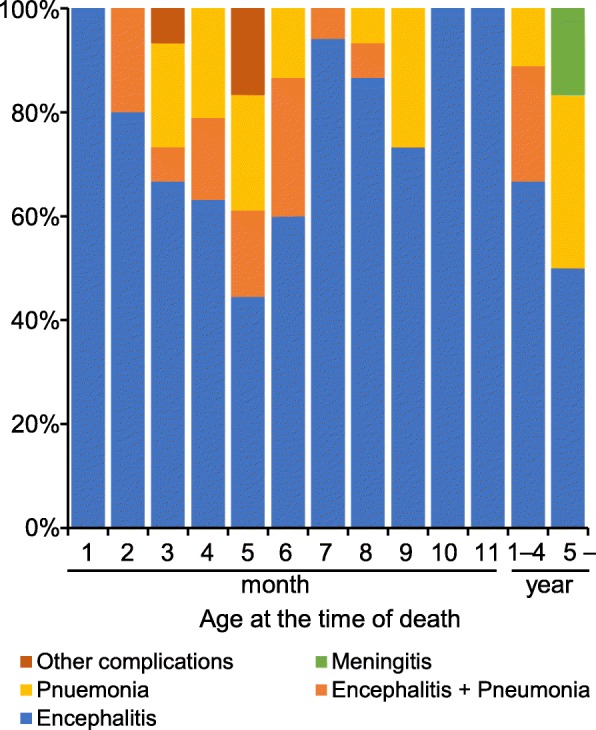
Complications of death cases (*n* = 140). Complications of measles-related death cases are categorized into five groups: encephalitis, B05.0; encephalitis + pneumonia, B05.0 and B05.2, J18.0 or J18.8; pneumonia, B05.2; meningitis, B05.8; and others, B05.8. The number of death cases with pneumonia (encephalitis + pneumonia, pneumonia) are significantly higher in children aged 0–6 months than those in the other age groups (*P* < 0.05; OR, 2.4; 95% CI, 1.9–5.3)

Encephalitis was the primary complication of measles-related deaths (*n* = 114 out of 140, Fig. 6). In addition, the number of death cases with pneumonia (encephalitis + pneumonia, pneumonia) was significantly higher in children aged 0–6 months (34.2%) than those in the other age groups (17.9%, *P* < 0.05).

## Discussion

This study reported the largest nationwide measles outbreak in Mongolia that occurred during 2015–2016 with two distinct waves. The duration of the outbreak was approximately two years, longer than the previous outbreak period, and a significantly larger number of cases were reported. In total, 53,737 measles cases and 140 measles related-deaths were reported during this period. The most affected age groups were children aged < 1 year and those aged 15–24 years. In 2015, Umnugovi aimag, the border province in China and the tourist destinations, and Ulaanbaatar city reported the highest attack rates. In 2016, 20 out of 21 provinces reported higher attack rate per 10,000 population than the previous years.

The first laboratory confirmed case was reported in March 2015. Since then, the reported case gradually increased and the first peak of outbreak occurred in June 2015. However, the dramatic downward trend was observed in July 2015, an upward trends were observed again from October 2015 and peaked again in March 2016, which coincided with the seasonal influenza in Mongolia that reached its peak from December to February [24]. This condition may have contributed to the measles transmission in hospital throughout Mongolia due to nosocomial infection. Previous studies also suggested that the climate factors significantly effect on the incidence of measles [25, 26].

In 2015, Ulaanbaatar city reported 19,432 (82.8%) cases (139 per 10,000 population), and Umnugovi aimag reported 494 (2.1%) cases (80 per 10,000 population). In 2016, an increasing trend in the incidence of new cases was reported across Mongolia. Almost all of border provinces of China and Russia were reported to have higher number of incidence cases per 10,000 population in 2016. Among them, Darkhan-Uul aimag reported the highest incidence (219 per 10,000 population), which was nearly double the incidence rate in Ulaanbaatar city in 2016. Darkhan-Uul aimag is the third highest populated aimag where major business activities are carried out in Mongolia. However, > 50.0% (15,965 cases) of confirmed cases in 2016 were reported from Ulaanbaatar city, which remained the highest number of reported case in 2016.

The high density in rural areas increases contacts among inhabitants, making favorable condition for disease outbreak [27]. Among all the aimags, Zavkhan with 70,546 population had a low incidence (8.6 per 10,000) in 2015 and the lowest (24.9 per 10,000) in 2016. Although the reason is unclear, this may be Zavkhan aimag is a mountainous area, where people reside sparsely.

Measles-related deaths and complications were more common among infants. Most of the death cases (125 out of 140 cases) were reported among children aged < 1 year than those who had not reached the eligible vaccination age. During the outbreak period, especially in the influenza season, a considerable number of infants visited health facilities during the second wave of outbreak due to influenza-like symptoms. Many parents confused the symptoms of measles as that of influenza, and these suspected cases might have been infected at the health facilities while waiting to receive medical treatment. Studies in Mongolia and Korea reported that nosocomial infection was one of the key risk factors during the measles outbreak [28, 29]. Then Mongolia government needs to consider preventive measures against nosocomial infection to prevent further outbreaks.

The WPRO reported on May 5, 2016, described that the first measles case on March 18, 2015 had a similar measles virus genotype as that circulating in China [30]. In 2015, Umnugovi aimag bordering China and Ulaanbaatar with an international airport had the highest attack rate. A measles outbreak was reported in the Inner Mongolia Region of China in 2014 [17]. In 2016, Bayan-Ulgii, Khovd and Uvs in the western region of Mongolia had the high attack rate. Kazakhstan had measles outbreak in 2015–2016 [31]. A recent study also reported that measles virus may be imported during the lunar new year in February, 2015, when mass border population movement occurred [28]. However, an epidemiological study assessing measles genotype by PCR would be necessary to reveal imported measles from neighboring countries.

The most affected age groups were children aged < 1 years and 15–24 years. A similar pattern was observed in the 2001 outbreak in Mongolia [20]. Measles outbreak among those aged 15–24 years might be explained by the accumulation of susceptible persons and/or their low immunity status caused by low-quality vaccines, disruption of vaccine cold chain, poor supply, and never vaccinated. The immunity gap can lead to measles outbreaks that highlighted the importance of a herd immunity and targeted vaccination of the susceptible populations [32]. Another possible reason may be misdiagnosis of measles. Since majority of medical professionals were aware that measles had been eliminated in Mongolia, they might have hesitated to diagnose and report measles cases before March 18, 2015, which might lead to further outbreak.

Measles cases living in Ulaanbaatar city had less risk for measles-related death; shortage of resources for medicine was speculated to contribute to the high risk in aimags. Thus, detailed factors, including immunization status, medical facilities, accessibility to health care and human resources, should be considered.

The Mongolian government implemented the supplementary immunization activities (SIAs) in children aged six months to six years from May to June in 2015 and in those aged 18–30 year in May, 2016. SIAs were performed on 371,971 people in 2015, covering 94% of targeted population and 549,846 covering 88% in 2016 during the SIA programs. However, the upward trend of the incidence rate and large numbers of cases continued to occur in aimags where SIA program was implemented in 2016.

The measles vaccine was first introduced in 1973, and the nationwide coverage was reported to be > 95% since 2005. Nevertheless, this outbreak demonstrates the importance of good quality vaccination programs in the control and prevention of communicable diseases. In addition to the routine vaccination, susceptible people should be vaccinated [33]. Those aged 15–24 years were born during the transition period in 1990–2000 after the separation from the Soviet Union, when the country experienced severe economic decline [34]. Many people from rural provinces were forced to move to cities to find jobs. The increased domestic migration during the transition period resulted in an increase in unregistered people, whose children were consequently excluded from the immunization program. The absence of a proper database of immunized people based on the citizens’ registry is another important issue to be resolved in the future.

The study has several limitations. First, the information was derived from the reported cases in the surveillance system. Misclassification and underreported cases could be presented although measles can be clinically diagnosed based on its signs and symptoms. Since the majority of medical professionals were aware that measles had been eliminated in Mongolia, they might have hesitated to diagnose and report measles before the Mongolian government officially announced its registry.

Although there might have been unreported cases, this dataset was the largest in Mongolia. The accuracy of information on reported cases was also dependent on the accuracy of reports submitted and stored on the surveillance system. Second, linkages to medical charts and death certificate of each case could not be confirmed. The details of clinical findings were not available in the dataset. Third, the vaccination status of patients was reported based on the vaccination booklet or patients’ response to questions asked by medical doctors at the onset of the disease. Thus, patients’ vaccination status cannot be validated. Accordingly, detailed analyses according to the vaccination status were not conducted. Despite these limitations, this study was the first report describing the measles outbreak in Mongolia during 2015–2016, with the permission from the Ministry of Health in Mongolia.

## Conclusions

This study reported the historical nationwide measles outbreak in Mongolia in post meals elimination era. In total, 53,737 cases including 140 deaths were reported in 2015–2016. The highest incidence rate were reported from Ulaanbaatar city, and Umnugovi aimag in 2015, and Darkhan-Uul aimag in 2016. The most affected age groups were children aged < 1 year, 15–24 years, and 25–34 years old. Despite the high overall vaccination coverage, accumulated susceptible clusters seemed to cause this measles outbreak. A systematic vaccination strategy is required to prevent another measles outbreak in Mongolia.

## Additional file

Additional file 1: Population data in Mongolia, 2015 and 2016. (PDF 175 kb)

## Abbreviation

CHD: Center for Health Development
EPI: Expanded Program on Immunization
MCV: Measles containing vaccine
MMR: Measles-mumps-rubella vaccine
SPSS: Statistical Package for the Social Sciences
WHO: World Health Organization
WPRO: WHO Regional Office for the Western Pacific
