# Supplementary material for: Epidemiological characteristics and trends of a Nationwide measles outbreak in Mongolia, 2015–2016
Source: BMC Public Health. 2019 Feb 15;19:201. doi: 10.1186/s12889-019-6511-0 (PMC6377723; doi:10.1186/s12889-019-6511-0)
Supplement: Supplementary file 1 — Population data in Mongolia, 2015 and 2016. (PDF 175 kb) [file 12889_2019_6511_MOESM1_ESM.pdf]

Additional file 1

Population data in Mongolia

31th December, 2015

	0 - 11 months	1 – 4 years	5 – 9 years	10 – 14 years	15 – 19 years	20 – 24 years	≥ 25 years	All age
Aimag								
Ulaanbaatar	38,144	145,917	130,587	84,732	90,873	110,522	744,725	1,345,500
Arkhangai	2,294	8,729	8,980	7,507	8,802	8,360	47,088	91,760
Baynkhongor	2,087	8,549	8,335	6,730	8,002	7,756	42,144	83,603
Bayn-Ulgii	2,715	10,247	11,082	10,354	9,829	9,857	43,703	97,787
Bulgan	1,392	5,396	5,683	4,329	5,018	5,080	32,754	59,652
Darkhan-Uul	2,413	9,387	9,494	6,772	7,755	8,418	53,452	97,691
Dornod	1,896	7,637	7,638	5,529	5,764	6,659	41,265	76,388
Dornogovi	1,704	6,441	6,630	4,960	4,935	5,305	33,150	63,125
Dundgovi	1,124	3,693	4,315	3,667	3,989	3,819	23,571	44,178
Govi-Altai	1,253	5,053	5,652	4,997	5,345	4,886	28,832	56,018
Govi-Sumber	433	1,838	1,749	1,219	1,309	1,340	8,420	16,308
Khentii	1,756	6,852	7,169	6,077	6,157	6,252	38,076	72,339
Khovd	2,181	8,773	8,802	7,575	8,547	7,922	39,327	83,127
Khuvsgul	3,108	12,487	12,907	10,232	11,068	11,603	66,018	127,423
Orkhon	2,401	9,511	9,277	6,684	7,478	8,757	54,642	98,750
Selenge	2,441	9,660	10,325	7,964	8,542	9,048	56,745	104,725
Sukhbaatar	1,567	5,829	5,598	4,434	4,954	5,251	31,159	58,792
Tuv	2,226	8,701	8,635	6,497	7,218	7,665	48,823	89,765
Umnugovi	1,670	6,281	6,013	4,693	5,120	5,282	32,308	61,367
Uvs	2,077	8,137	8,977	7,536	8,181	7,870	37,618	80,396
Uvurkhangai	2,904	10,493	11,230	9,158	10,615	9,914	57,593	111,907
Zavkhan	1,661	6,133	7,369	5,761	6,531	6,168	36,018	69,641
All Aimag	79,447	305,744	296,447	217,407	236,032	257,734	1,597,431	2,990,242

31th December, 2016

	0 - 11 months	1 – 4 years	5 – 9 years	10 – 14 years	15 – 19 years	20 – 24 years	≥ 25 years	All age
Aimag								
Ulaanbaatar	33,211	146,295	140,362	86,704	88,152	104,399	781,669	1,380,792
Arkhangai	2,002	8,948	9,533	7,328	8,874	8,372	48,452	93,509
Baynkhongor	1,781	8,625	8,963	6,580	7,945	7,656	43,782	85,332
Bayn-Ulgii	2,378	10,586	11,306	10,003	9,784	9,866	45,423	99,346
Bulgan	996	5,534	6,048	4,328	5,060	5,233	33,950	61,149
Darkhan-Uul	2,088	9,681	10,130	6,835	7,622	8,100	55,340	99,796
Dornod	1,844	7,746	8,278	5,604	5,793	6,352	42,155	77,772
Dornogovi	1,385	6,735	7,123	5,180	5,155	5,302	35,726	66,606
Dundgovi	1,029	4,003	4,459	3,625	4,016	3,975	24,408	45,515
Govi-Altai	1,025	5,075	5,837	4,886	5,472	4,810	29,589	56,694
Govi-Sumber	414	1,929	1,850	1,295	1,331	1,358	8,757	16,934
Khentii	1,683	7,057	7,587	5,913	6,242	6,200	38,926	73,608
Khovd	1,830	8,819	9,475	7,471	8,486	8,305	40,939	85,325
Khuvsgul	2,465	12,741	13,730	10,219	11,075	11,476	68,251	129,957
Orkhon	2,073	9,732	10,225	6,576	7,427	8,661	57,197	101,891
Selenge	2,034	9,778	10,995	8,039	8,468	8,541	58,822	106,677
Sukhbaatar	1,257	5,929	6,157	4,443	4,940	5,059	32,247	60,032
Tuv	1,781	8,957	9,423	6,551	7,266	7,560	51,294	92,832
Umnugovi	1,428	6,612	6,674	4,598	5,215	5,238	33,896	63,661
Uvs	2,027	8,150	9,302	7,305	8,059	7,889	38,645	81,377
Uvurkhangai	2,412	10,899	11,712	9,019	10,530	10,002	59,337	113,911
Zavkhan	1,436	6,370	7,463	5,686	6,641	6,224	37,032	70,852
All Aimag	68,579	310,201	316,632	218,188	233,553	250,578	1,665,837	3,063,568
